# Multiplexing Bioluminescent and Fluorescent Reporters to Monitor Live Cells

**DOI:** 10.2174/1875397300801010011

**Published:** 2008-02-25

**Authors:** Michael Haugwitz, Omar Nourzaie, Tatiana Garachtchenko, Lanrong Hu, Suvarna Gandlur, Cathy Olsen, Andrew Farmer, Grigoriy Chaga, Hiroaki Sagawa

**Affiliations:** a Clontech Laboratories, Inc., 1290 Terra Bella Ave., Mountain View, CA 94043, USA; b Molecular Devices, now a part of MDS Analytical Technologies, 1311 Orleans Drive, Sunnyvale, CA 94089, USA

## Abstract

Reporter proteins are valuable tools to monitor promoter activities and characterize signal transduction pathways. Many of the currently available promoter reporters have drawbacks that compromise their performance. Enzyme-based reporter systems using cytosolic luciferases are highly sensitive, but require a cell lysis step that prevents their use in long-term monitoring. By contrast, secreted bioluminescent reporters like *Metridia* luciferase and Secreted Alkaline Phosphatase can be assayed repeatedly, using supernatant from the same live cell population to produce many sets of data over time. This is crucial for studies with limited amounts of cells, as in the case of stem cells. The use of secreted bioluminescent reporters also enables broader applications to provide more detailed information using live cells; for example, multiplexing with fluorescent proteins. Here, data is presented describing the characteristics of secreted *Metridia* luciferase and its use in multiplexing applications with either Secreted Alkaline Phosphatase or a fluorescent protein.

## INTRODUCTION

The regulation of cellular gene expression *via *activation and inactivation of a network of signal transduction pathways enables cells to grow, differentiate, and communicate with each other and their surroundings, as well as to react to other changes in their environment [1].

These crucial events in cell biology are of great interest for many types of cell studies. For example, in the case of stem cell-related research a specific set of transcription factors, Oct4, Sox2, c-myc, and Klf4, has been found to be sufficient to reprogram fibroblasts into pluripotent-like stem cells [2, 3]. These results emphasize the powerful effect of promoter regulation *via *transcription factors.

In order to enable further discoveries in the field of signal transduction, it is crucial for cell biologists to be able to monitor promoter activation *via *a reliable, easy to measure, and consistent reporter system. To date, a variety of reporter systems have been developed, all of which rely on the expression of a reporter-encoding gene driven by the promoter of interest. Reporter detection can then be correlated with promoter activation.

The reporter itself, and its method of detection, vary between the different assays. Reporter activity is typically measured either by the fluorescence intensity of the reporter itself or by detection of a specific enzymatic activity of the reporter. Examples of fluorescent reporters include *Aequorea victoria*-based GFP variants [4], *Aequorea coerulescens-*based AcGFP [5], and *Anthozoa-*based fluorescent reporters including ZsGreen and DsRed variants [6]. One advantage of fluorescent protein reporters is that detection does not rely on any cofactors or exogenous substrates. Rather, the fluorescent signal results from the intrinsic chromophore structure. This enables researchers to design completely homogeneous assays to monitor gene expression continuously, on a cell by cell basis.

There are two potential disadvantages to using fluorescent reporters. The first is that unfortunately, most fluorescent proteins have slow turnover rates, which cause the reporter to accumulate over time. This is problematic if the goal is to monitor fast activation and inactivation of a promoter of interest. Destabilized fluorescent proteins, which have a drastically accelerated turnover due to a proteasome targeting sequence fused to the fluorescent reporter protein [7], have been developed to address this problem. These destabilized fluorescent proteins have a shorter life span and are indeed able to reflect fast up- and down-regulation of the promoter. A second potential disadvantage of fluorescent reporters is that the signal generated by fluorescent proteins is not based on an enzymatic, amplifying reaction. Therefore, the sensitivity of these assays is lower compared to enzyme based reporter assays.

The most commonly used enzyme based assays are bioluminescence based luciferase assays. Luciferases are oxygenases; that is, they use oxygen together with their respective substrate to produce a bioluminescent signal [8, 9].

Cytosolic luciferase reporters such as Firefly and *Renilla* luciferase fall into two classes. The first class, which includes Firefly luciferase, requires both luciferin and ATP as substrates in order to produce a luminescent signal. It has been used for a variety of assay applications [10], including assays designed to detect cytosolic ATP levels (e.g., apoptosis and cytotoxicity assays) [11]. The second class, which includes *Renilla* luciferase and several other luciferases, use coelenterazine as their substrate and do not require ATP [12].

Both classes of luciferase are very sensitive and have broad dynamic ranges. However, the cells must be lysed in order to allow the substrate to access these cytosolic luciferase reporters. Therefore, unlike fluorescent proteins, cytosolic luciferases do not permit the development of live cell assays and the researcher is limited to a single data point per experiment. This is a serious disadvantage, especially if access to specific cells is limited or if long term monitoring of the same cells is the main purpose of the experiment (as in time course, differentiation, or toxicity studies). However, some new membrane permeable luciferase substrates have been developed, which allow the detection of luciferase activity inside the cell without the requirement for cell lysis.

An additional drawback is that the bioluminescent signal detected from luciferases only indicates the total quantity of reporter expressed by the cell population as a whole. It does not reveal if the majority of sampled cells are expressing the reporter, or only a subset.

Previous attempts to solve this problem have included creating secreted variants of *Renilla* luciferase tagged with a secretion signal peptide sequence. *Renilla* activity was detectable in the media supernatant; however, the specific activity of the secreted form of *Renilla* luciferase was approximately 10-fold less than that of cytosolic *Renilla* luciferase when measured in mammalian cells. This may be due in part to cysteine residues in the *Renilla* luciferase sequence which can be oxidized in the secretory pathway environment, compromising the enzymatic activity of the secreted protein. The detection limit for secreted *Renilla* luciferase in cell media was 100 fg [13].

Another approach is to avoid this problem by using naturally secreted proteins which are inherently optimized for proper folding and structure in the oxidizing environment of the secretory pathway. One such example is secreted alkaline phosphatase (SEAP) [14]. This protein has already been successfully used as a promoter reporter in a variety of applications [15, 16] including effective use in transgenic animals for long-term monitoring of ovarian tumor growth and response to therapeutics [17].

SEAP does have disadvantages, in that most cell culture media samples and blood samples contain naturally occurring serum alkaline phosphatase which can interfere with the detection of the reporter molecule SEAP. Fortunately, SEAP is heat resistant, so samples can be heat treated to inactivate serum alkaline phosphatase before the actual reporter SEAP is measured. However, the requirement for a heat inactivation step limits throughput and can be especially problematic for screening assays. The heat inactivation step can be avoided by using serum free medium. This allows immediate measurement of SEAP activity in the sample without the requirement for heat inactivation.

More recently, naturally secreted luciferases have been cloned and characterized. Since there is no luciferase naturally present in serum, these secreted luciferases can be measured immediately after substrate addition, without an intervening heat inactivation step.

Secreted *Gaussia* luciferase (*Gaussia princeps*) [18] and secreted *Metridia* luciferase (*Metridia longa*) [19] have been cloned from members of the same family of *Crustacea*. Both use coelenterazine as their substrate. *Gaussia *luciferase has been shown to emit light with a flash-like bioluminescence characteristic [18]. *Metridia* luciferase has a more “glow-like” bioluminescence characteristic. Its signal stability is about 10 times greater than that of *Gaussia* luciferase (data not shown).

All secreted reporter have the intrinsic advantage of not relying on a fast turnover rate, whereas cytosolic reporters do, in order to guarantee a low background. Any secreted reporter that accumulates in the media before the actual experiment, for example due to promoter leakage, can easily be removed by changing the media just before starting the actual experiment. This can not be done with cytosolic reporters. Secreted reporter actually benefit from their stability, because a larger amount of the reporter can accumulate in the media over the course of the experiment ensuring a larger dynamic range.

Secreted reporters enable applications including time course experiments, long term monitoring during differentiation, repeated induction, and other experiments which cannot be done in an efficient manner using cell lysis-based assays. Multiplexing secreted reporters, like *Metridia* luciferase and SEAP, offers additional advantages, such as the ability to monitor two signaling pathways simultaneously or to use one of the two reporters as a control for expression and the integrity of the secretory pathway. This multiplex capability is especially important when using secreted reporters to screen chemical compound libraries.

However, even though a secreted reporter system allows for live cell monitoring, it is still limited to use as a cell population assay. Therefore combining a live-cell population assay using a secreted reporter for quick and easy assay sampling, with a live cell by cell assay which uses fluorescent proteins as described above to capture more precise, cell-based data points, can be an even more powerful method.

This study provides data which establishes secreted *Metridia* luciferase as a very useful reporter for single reporter assays and as a multiplex partner with another secreted reporter, SEAP. In addition, data is presented to support a new strategy for developing multiplex assays which combine the advantages of a bioluminescent secreted reporter system with those of a fluorescent protein based “cell by cell” assay.

This combination helps to close the gap between High throughput screening (HTS) and high content screening (HCS) approaches.

## MATERIALS AND METHODOLOGY

### Materials

Ready-To-Glow™ Secreted Luciferase Reporter Assay kit containing the Vector Set, Substrate, Substrate Dilution Buffer and Reaction Buffer (Clontech, Mountain View, CA). The cDNA sequence encoding secreted *Metridia* luciferase used in the vectors set has been human codon-optimized, potential mRNA splicing sites have been removed and the GC content has been increased to ensure a higher mRNA stability; Great EscAPe™ SEAP Chemiluminescence Kit 2.0 (Clontech, Mountain View, CA); Living Colors® HEK 293 ZsGreen Proteasome Sensor Cell Line (Clontech); TALON® Express Bacterial Expression and Purification Kit (Clontech, Mountain View, CA); Bacteria strain BL21(DE3) Stratagene; DMEM (Sigma); Tet System Approved Fetal Bovine Serum (Clontech); Cell lysis buffer from *Renilla* luciferase kit (Promega); SDS-PAGE (Biorad); FuGENE® 6 (Roche); TNF-α (Clontech, Mountain View, CA); ALLN (Sigma); Forskolin (Sigma); Black Walled, Clear Bottom 96-well Plates (Costar).

### Recombinant *Metridia* Luciferase

The *Metridia* luciferase gene, lacking the 5’ coding region for the first 17 amino acids (secretion signal), was cloned into the pEcoli-N term 6xHN (Clontech) and pEcoli-C term 6xHN (Clontech) vectors. Both vectors use a T7 promoter to drive the expression of the *Metridia* luciferase gene.

The constructs were transformed into BL21(DE3) (Stratagene). A positive clone of each construct was then cultured in 200 ml of LB Miller for 16 hours without induction.

The recombinant protein was extracted using 6 M Urea with 0.75 mg/ml of lysozyme in the extraction buffer. The resuspended pellet was sonicated for 1 minute on ice and then rested on ice for 2 minutes. This sonication cycle was repeated another four times. The protein was then purified using Talon resin (Clontech) following the standard batch purification protocol. All the buffers in the kit, including the elution buffer, had 6M Urea added to them. A TALON resin bed volume of 1 ml was used. The recombinant protein was eluted using 3 ml of 1X elution buffer.

After elution, the protein was dialyzed down stepwise, reducing the concentration of Urea from 6 M, to 4 M, 2 M, 1 M, and finally 1X PBS. Each buffer exchange involved 16 hours of exchange at 4°C, at a volume of 2 L.

The protein concentration of the final material was determined *via *the BCA method (Pierce). The samples were also run on an SDS-PAGE gel (Bio-Rad) and stained with Coomassie Blue (Bio-Rad) to verify the purity of the recombinant protein.

### Detection Limit and Dynamic Range of Recombinant *Metridia* Luciferase

The purified *Metridia *luciferase was spiked into DMEM tissue culture media containing 10% Tet System Approved FBS (Clontech) at concentrations of 1 fg to 1 x 10^6^ fg per well in a 96-well plate. To detect *Metridia* luciferase activity using the Ready-To-Glow Secreted Luciferase Reporter Assay kit (Clontech) (see Materials), 50 µl of substrate-reaction buffer mix (0.5 µl of substrate and 45.5 µl of reaction-buffer) was added to each sample. *Metridia *luciferase activity was measured immediately after addition of substrate using the SpectraMax® L plate reader (Molecular Devices).

### Secretion Efficiency

Each well of a 6-well plate was seeded with 1.2 x 10^5^ CHO-K1 (ATCC) cells. 24 hours later, the cells were transiently transfected using the FuGENE 6 (Roche) transfection reagent. One µg of DNA (pMetLuc-Control, Clontech), which expresses secreted luciferase under a constitutive CMV promoter, was used per well, and cells were transfected from one transfection mix. At specified time points after the actual transfection, the total supernatant (2 ml) was collected and mixed with 0.5 ml of cell lysis buffer. The cells in each corresponding well were lysed using 0.5 ml cell lysis buffer (Promega). Then 2 ml of DMEM media +10% FBS was added to the lysate in order to ensure comparable sample composition between each lysate and its corresponding supernatant. The samples were assayed by adding 5 µl of substrate-reaction buffer mix to 50 µl of sample (“Media” and “Lysate”) and analyzed on the BD Monolight™ 3096 Microplate Luminometer (BD Biosciences Pharmingen).

### Secreted *Metridia* Luciferase Promoter Reporter Assay

The NFκB response element was cloned into the multiple cloning site of the pMetLuc-Reporter vector (Clontech) upstream of the *Metridia* luciferase gene. The accuracy of the construct was determined by DNA sequencing. HeLa cells (ATCC) were transiently transfected with the pNFkB-MetLuc construct in a 10 cm plate (~5 x 10^6^ cells), using FuGENE 6 (Roche) and 10 µg of DNA. Twelve hours post-transfection, cells were split into 6 cm dishes with a total volume of 2 ml of DMEM + 10% FCS. Twelve hours post-split, the media from the two plates was replaced with either 2 ml of new DMEM + 10% FBS without TNF-α (negative control) or with 2 ml of DMEM media +10% FBS containing 100 ng/ml TNF-α (Clontech). Supernatant from the plates were collected 6 hours later. The samples were assayed by the addition of 5 µl of Ready-To-Glow substrate-reaction buffer mix to 50 µl of supernatant and analyzed on a Veritas™ Microplate Luminometer (Turner BioSystems). Signal intensities of the samples treated with and without TNF-α were obtained 1 minute, 10 minutes, and 30 minutes after addition of substrate and used to calculate corresponding “fold increase” values.

### Multiplexing Secreted *Metridia* Luciferase with SEAP

HeLa cells (ATCC) were transiently co-transfected with pNFkB-MetLuc (see above) and pCRE-SEAP (Clontech) constructs in a 10 cm plate, using FuGENE 6 (Roche) and 5 µg DNA per construct (10 µg total DNA). Twelve hours posttransfection, the cells were split into 6-well plates in a total volume of 2 ml of DMEM with 10% FBS each. Twelve hours post-split the media from the plates was replaced with either 2 ml of DMEM with 10% FBS (negative control) or 2 ml of DMEM + 10% FBS containing either 100 ng/ml TNF-α (Clontech), or 2ml of DMEM + 10% FBS containing 10 µM Forskolin (Sigma). Total supernatant from the plates was collected 6 hours later. Samples for the secreted luciferase assay were prepared by adding 5 µl of Ready-To-Glow substrate-reaction buffer mix to 50 µl of supernatant. For the SEAP assay (Great EscAPe SEAP Chemiluminescence Kit 2.0, Clontech), 25 µl of supernatant volume was diluted with 75 µl of 1X dilution buffer and heat inactivated at 65°C for 30 min. The sample was equilibrated to room temperature prior to addition of 100 µl substrate. All samples, luciferase and SEAP, were read using the BD Monolight 3096 Microplate Luminometer (BD Biosciences Pharmingen).

### Multiplexing Secreted *Metridia* Luciferase with ZsGreen Proteasome Sensor Cell Line

The Living Colors® HEK 293 ZsGreen Proteasome Sensor Cell Line (Clontech) was transiently transfected with the pNFκB-MetLuc construct (see above) in a 10 cm plate, using FuGENE 6 (Roche) and 10 µg of DNA. Twelve hours posttransfection, 3 x 10^4^ cells were seeded into each well of a black-wall, clear-bottom 96-well plate (Costar). Sixteen hours post-split the media from the plates was removed and replaced with either 200 µl of DMEM + 10% FBS (negative control), 200 µl of DMEM + 10% FBS containing 25 ng/ml TNF-α (Clontech), 200 ml of DMEM + 10% FBS containing 50 µM ALLN (Sigma), or 200 µl of DMEM + 10% FBS containing both 25 ng/ml TNF-α and 50 µM ALLN. Sixteen hours posttreatment, 50 µl of Ready-To-Glow substrate-buffer mix (0.5 µl substrate and 45.5 µl reaction buffer) was added to each well. The luminescence and fluorescence signal (Ex. 493 nm/Em. 520 nm with a 515 nm cut-off) of each well was measured consecutively using the SpectraMax® M5 plate reader (Molecular Devices).

## RESULTS

### Detection Limit and Dynamic Range of Recombinant *Metridia* Luciferase

The major enzymatic characteristics of secreted *Metridia* luciferase regarding signal stability after addition of substrate, detection limit, and dynamic range, were studied using recombinant, 6xHN tagged *Metridia* luciferase. The recombinant protein was expressed in *E. coli* without the N-terminal 17 amino acid long secretion signal to allow for a higher expression yield, and purified on TALON resin as described above.

**Fig. (1):**
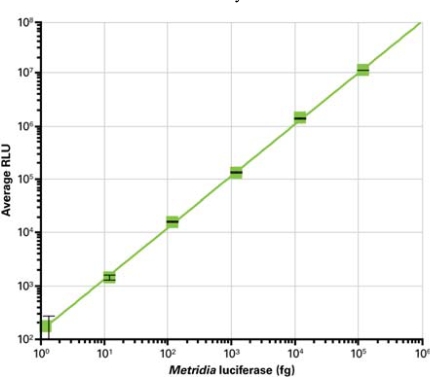
Detection limit and linear range of recombinant Metridia luciferase activity. Various amounts of recombinant Metridia luciferase were spiked into DMEM + 10% FBS in a 96-well plate. After addition of substrate, the plate was analyzed using a Molecular Devices SpectraMax® L plate reader.

In order to gain data relevant for the use of *Metridia* luciferase in standard mammalian cell culture conditions, various amounts of purified protein were spiked into DMEM containing 10% FBS. The results show a high level of sensitivity for the *Metridia* luciferase reporter enzyme. Upon addition of coelenterazine substrate, it was possible to detect as little as 40 fg of recombinant *Metridia* luciferase per ml, or 2 fg per well of a 96-well plate**.** This corresponds to approximately 46,000 molecules (Fig. **1**).

The linear range of *Metridia* luciferase activity was determined by serial dilution of the recombinant protein, followed by addition of substrate. This experiment showed that *Metridia* luciferase activity is linear over a range of at least 6 logs (Fig. **1**).

In order to determine the decay rate of the bioluminescent signal from recombinant *Metridia* luciferase, substrate was added and the signal was measured at different time points after the addition of substrate.

Ten minutes after the initial addition of substrate, the signal decreased to 50% of its original strength (data not shown). This signal decay is much slower than it has been shown for another secreted luciferase, from *Gaussia princeps*. For *Gaussia* luciferase, the signal dropped to only 25% of its original intensity within about 50 seconds after addition of substrate, and to just 10% of its original intensity 40 seconds later [18]. This shows that in comparison to *Gaussia* luciferase, *Metridia* luciferase can be categorized as a “glow” luciferase and not as a “flash” luciferase.

### *Metridia* Luciferase is a Well-Secreted Reporter Enzyme

*Metridia* luciferase contains an endogenous secretion signal, and is a naturally secreted protein of *Metridia longa*. Its folding and secretion efficiency is likely to be optimal in the environment of the endoplasmic reticulum and the downstream compartments of the secretory pathway. Due to the evolutionarily conserved machinery and requirements of the secretory pathway which span organisms from yeast to mammalian cells [20, 21], *Metridia* luciferase is also likely to be well secreted in other eukaryotic cells.

To investigate this hypothesis, the constitutive expression and secretion of *Metridia* luciferase in CHO cells was monitored over time using a strong constitutive promoter (CMV). Using this promoter, it was possible to challenge the expression, ER folding and secretion machinery of the cells.

**Fig. (2):**
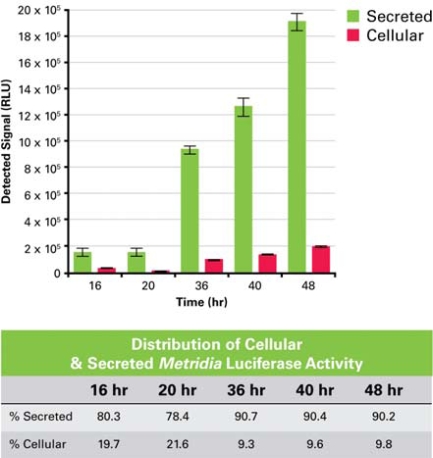
Signal intensity of *Metridia* luciferase measured in either supernatant (secreted) or lysate (cellular) over time.

At different time points after the initial transient transfection with the constitutive expression construct, the supernatant and the corresponding cells were harvested, and the activity of the enzyme in the supernatant and in the cell lysate was tested by addition of substrate. The first sample, taken 12 hours after the initial transfection, showed equal signal intensity in the supernatant and the cell lysate samples (data not shown). However, in the following time periods (up to 48 hours posttransfection), the percent activity from *Metridia* luciferase remaining inside the cells dropped to just 9% of the total and remained relatively steady, with 91% of the reporter activity detected in the media supernatant (Fig. **2**). The fact that the relative amount of *Metridia* luciferase in the cell lysate does not increase over time, and in fact decreases, is a good indication that the reporter is a well-secreted protein. However, a complete picture regarding its secretion efficiency can only be obtained by establishing a stable cell line expressing secreted *Metridia* luciferase. This will allow a comparison between the ratios of intracellular versus extracellular levels of *Metridia* luciferase and intracellular versus extracellular levels of an endogenous secreted protein

We also found the *Metridia* luciferase itself to be a very stable protein (data not shown). Once secreted into the culture media of cells, the protein is stable for at least 72 hours at 37°C without a significant loss of activity. This allows the accumulation of the reporter over a long time period without any loss of reporter molecules *via *degradation. The accumulation ensures a much larger dynamic range over a longer period of time. This means that the majority of reporter molecules synthesized and secreted during the experiment will contribute to the bioluminescent signal. In contrast, cytosolic reporters like Firefly Luciferase have a functional half life of approximately 3–4.5 hours [22]. Therefore, only the reporter molecules present at the time of sample collection contribute to the actual bioluminescent signal, because the reporter molecules that were expressed earlier in the course of the experiment have been degraded in the meantime.

**Fig. (3):**
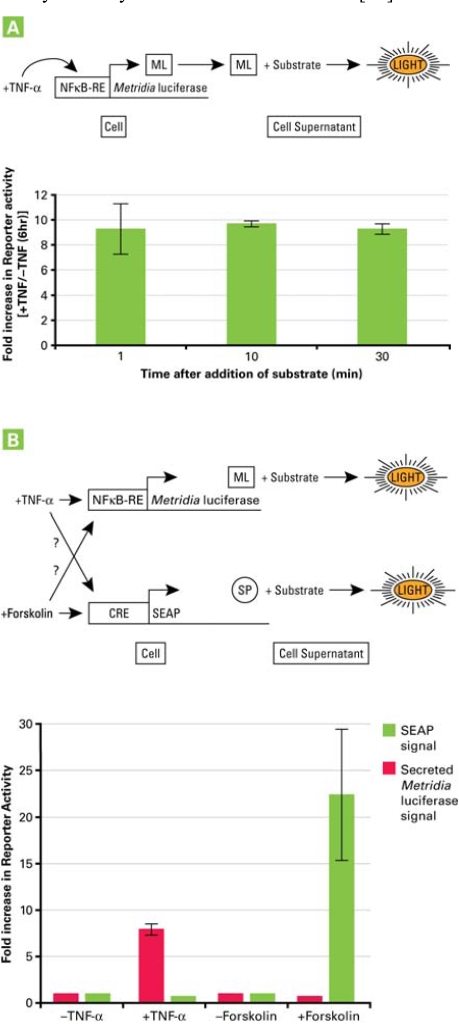
Use of *Metridia* luciferase in single or multiplexed applications with another secreted bioluminescence reporter. Panel **A**. Monitoring promoter activation using the sequence-optimized secreted *Metridia* luciferase reporter. HeLa cells were transiently transfected with a vector construct containing the NFκB response element driving the expression of sequence-optimized secreted *Metridia* luciferase. 24 hr after transfection, the media was removed and replaced by media with or without TNF-α  (100 ng/ml) to activate the NFκB signal transduction pathway. Six hr after addition of TNF-α, samples of the media were removed and analyzed for *Metridia* luciferase activity. The fold induction was calculated for different time points following the addition of substrate. Panel **B**. Monitoring the activities of two promoters using two secreted reporters, secreted *Metridia* luciferase and SEAP.

The use of secreted *Metridia* luciferase as a promoter reporter was tested using a reporter construct consisting of the NFκB response element cloned upstream of the humanized *Metridia* luciferase sequence. Twenty four hours after transfecting this construct into HeLa cells, the supernatant was removed and replaced with fresh culture medium with or without TNF-α for 6 hours. At that time, culture supernatant samples were collected and assayed for the presence of the reporter, by addition of the luciferase substrate. Either ten or thirty minutes after the addition of substrate, a stable 11-fold increase in bioluminescent signal was detected in the supernatant of cells treated with TNF-α in comparison to the signal detected in the supernatant of cells not treated with TNF-α (Fig. **3A**).

After the initial sampling, one could either sample the supernatant of the same set of cells at a later time point after TNF-α induction, or remove the medium and replace it with new media containing another inducer on exactly the same cells. The change of media would remove any residual secreted *Metridia* luciferase reporter, setting the background close to zero. Therefore, any *Metridia* luciferase signal detected in a media sample after the second treatment reflects the effect of the subsequent treatment, but not of the original TNF-α treatment.

### Multiplex Assay with Two Reporters

Multiplexing two different bioluminescent secreted reporters, using different substrates, would provide even more information on the cell state without sacrificing the cells and would create new advantages for experimental design and analysis.

For example, one of the two secreted reporters could be used as a transfection control for normalization purposes. Alternatively, it could be used as a positive, constitutive control to monitor the functionality of the secretory pathway itself. A third application would be monitoring two different signal transduction pathways at the same time. This would enable studies to examine potential cross-communication between intracellular signal transduction pathways. With regard to this possibility, we have used secreted *Metridia* luciferase and SEAP to answer the question of whether the c-AMP and NFκB signal transduction pathways are interrelated [14].

HeLa cells were cotransfected with two constructs: the first construct containing the NFκB response element driving the expression of humanized *Metridia* luciferase and the second construct containing the cAMP response element (CRE) and driving the expression of SEAP. 24 hours after transfection, the media was exchanged with fresh media containing either TNF-α (for NFκB) or forskolin (for CRE).

Six hours after treatment with the respective inducers, the supernatant was collected and tested for the presence of both reporters. The results showed that the NFκB and the CRE pathways are independent from each other and do not cross-communicate. This was shown by a specific activation of the NFκB pathway *via *TNF-α, indicated by the presence of secreted *Metridia* luciferase in the media supernatant without any increase in the presence of the second reporter, SEAP, in the media supernatant; and vice versa after treatment of transfected cells with forskolin, a specific inducer of the cAMP signal transduction pathway (Fig. **3B**).

### Live Cell Multiplexing of Bioluminescent and Fluorescent Protein Based Reporter Assays

In the past, multiplexed reporter assays have been restricted to the use of different bioluminescent assays or different fluorescent reporter based assays. However, new developments in plate reader design have made it possible to detect bioluminescent and fluorescent reporters at the same time, in the same well. These advances create new multiplexing opportunities combining fluorescent protein reporter assays and bioluminescent reporter assays, especially when the assays can be performed using live cells.

This broader range of reporter and measurement combinations greatly expands the potential of multiplex assays, unlimited by the method of detection and without the need to sacrifice the cells. Here we show a powerful example of such a multi-mode, multiplexing approach. We made use of a fluorescent protein based cell line, the Living Colors Proteasome Sensor HEK 293 Cell Line, which is based on the constitutive expression of a destabilized fluorescent protein, ZsProSensor-1. In this case, ZsGreen is expressed as a fusion protein with a domain that rapidly targets the protein for proteasomal degradation, resulting in a very low level of green fluorescence in the cytosol of the stable cell line, if the proteasomes are active. However, if the function of the cellular proteasomes is compromised, ZsGreen is not degraded and rapidly accumulates, yielding a sharp rise in the fluorescent intensity in the cytosol of this stable cell line [23].

This cell line was used to monitor the importance of proteasomal activity in the NFκB dependent, TNF-α induced signaling pathway.

It has been shown that phosphorylation of IκB alone upon TNF-α treatment is not sufficient to allow NFκB induced promoter activation [24, 25].

**Fig. (4):**
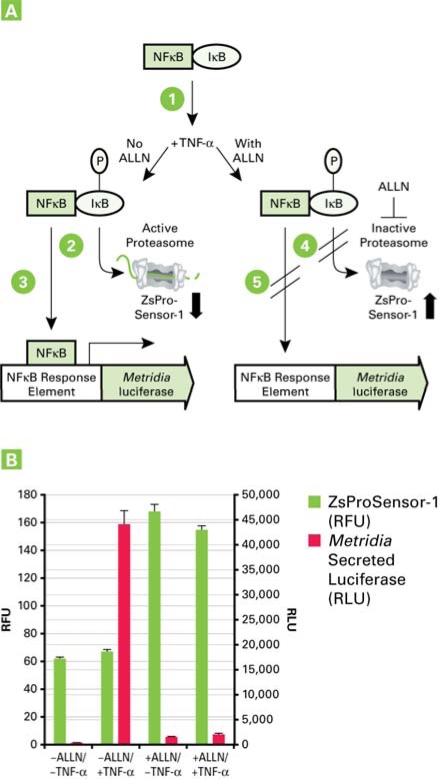
Panel A. Requirement for active proteasomes in TNF-α-induced, NFκB-dependent signaling. Inactive NFκB is sequestered in the cytoplasm by IκB. IκB must be phosphorylated upon TNF-α induction (1) and degraded by the proteasome (2) in order for NFκB to translocate to the nucleus and initiate signaling (3). Alternatively, when the proteasome is inhibited by the peptide ALLN (4), IκB is not degraded and NFκB cannot translocate (5). The status of the proteasome (active or inactive) can be monitored based on ZsProSensor-1 levels. Panel B. NFκB activation by TNF-α requires proteasomal activity. High levels of *Metridia* luciferase signal were only observed in the absence of ALLN and the presence of TNF-α. When the proteasome is inactivated by ALLN (monitored by increasing levels of ZsProSensor-1 fluorescence), the NFκB signaling pathway cannot respond effectively to TNF-α stimulation.

Consistent with these findings, our results show that phosphorylation of IκB must be followed by its proteasomal degradation, in order to allow NFκB-based promoter activation (Fig. **4A**).

In this experiment, the NFκB signal transduction pathway was monitored using the same NFκB-response element construct used earlier (Fig. **3**), which was transiently transfected into the Living Colors HEK-293 Proteasome Sensor Cell Line.

This allowed us to monitor TNF-α dependent activation of the NFκB pathway *via *a bioluminescent reporter, in a cell line that allows simultaneous monitoring of proteasome activity *via *a fluorescent protein-based readout. The result showed that TNF-α can only activate the NFκB signal transduction pathway in cells with active proteasomes. Inhibition of proteasomal activity, for example *via *ALLN (N-acetyl-leucyl-leucyl-norleucinal), caused a complete interruption of NFκB-dependent promoter activation (Fig. **4B**).

This experiment highlights the power of multiplexing bioluminescent and fluorescent reporters in a live cell assay, by allowing detection of two events from the same well of a 96-well plate simply by collecting both fluorescent and bioluminescent signal using a multi-mode plate reader.

## DISCUSSION

The data presented here demonstrate new opportunities in live-cell assay development, by combining secreted bioluminescent reporter assays with each other as well as by multiplexing them with fluorescent protein reporter assays using multi-mode plate readers.

With a very low limit of detection—as little as 2 fg of recombinant protein per well of a 96-well plate—and a broad linear range of 6 logs, the enzymatic characteristics of *Metridia* luciferase are comparable with those of other, non-secreted luciferase reporters as well as with another secreted chemiluminescent reporter, SEAP [26].

We have shown that the utility of *Metridia* luciferase as a secreted reporter is not compromised by the fact that it must pass through the secretory pathway in order to be secreted outside the cell. The protein is secreted efficiently and does not accumulate within the secretory pathway.

The ease of detecting a reporter *via *direct addition of substrate to the media supernatant of cells, without sacrificing the cells themselves is just one advantage of secreted reporter systems. An even greater advantage lies in the fact that this system can be multiplexed with other live cell assays, including other secreted reporter assays such as SEAP and even fluorescent protein assays.

This is particularly useful in screening applications, where compounds in a chemical library may interfere with the secretory pathway. This could lead to the identification of a false positive compound because the change in the detectable amount of *Metridia* luciferase in the media is not caused by an effect of the compound on the promoter of interest, but by targeting the secretory pathway itself. Using a cotransfected, constitutively expressed secreted reporter such as SEAP could act as a very important control for the proper function of the secretory pathway.

As we have shown here, it is possible to dissect two signal transduction pathways from each other by using the two secreted bioluminescent reporters *Metridia* luciferase and SEAP. We have clearly demonstrated that two signal transduction pathways, when induced by TNF-α and Forskolin respectively, are independent from each other. Although this result could also have been obtained using cytosolic bioluminescent reporter systems, the cell lysis required to gain access to the intracellular reporter would have made it impossible to obtain any additional data sets that required live cells or to perform long term monitoring of the identical cell population.

Toxicity assays are another example of a field where there would be a benefit to repeated data acquisition from live cells. Using a secreted reporter would make it possible to monitor the toxic effects of compounds or treatments over a prolonged exposure time. It would also allow the monitoring of potential drug interactions over time, dependent on the time as well as the order in which the drugs were administered.

We have also demonstrated that using a secreted reporter system allows for other, more innovative multiplexing applications by combining bioluminescent assays with fluorescent protein based assays utilizing more advanced, new generation plate readers. These plate readers offer the ability to simultaneously detect absorbance, fluorescence, luminescence, and other readouts.

We were able to combine secreted *Metridia* luciferase as a reporter for TNF-α induced NFκB activation, with the HEK-293 fluorescent protein based Proteasome Sensor cell line [23, 27]. Combining these assays made it possible to prove the requirement for proteasomal activity (monitored by an increase or decrease in green fluorescence intensity) in TNF-α dependent activation of the NFκB pathway, as described by Haas *et al.* and Sun *et al.* [24, 25]. It is important to note that the results from this experiment were obtained from the same well by simply changing between fluorescence and luminescence detection modes on the plate reader, increasing the amount of data that could be obtained simultaneously. In addition, the format of such a multiplexed assay saves a considerable amount of time and reduces the possibility of error due to additional handling.

Multiplexing secreted bioluminscent assays and fluoresecent protein based assays, particularly translocation assays, allows one to combine a “cell population” assay (for example, a secreted luciferase promoter reporter assay) with a “cell by cell” assay (for example, a fluorescent fusion protein translocation assay). In the alternative scenario, where the cells must be lysed in order to access to a cytosolic “cell population” reporter, the information that could have been gained from a fluorescent protein based translocation assay is lost.

Using a secreted reporter molecule as the multiplex partner eliminates this problem and makes it possible to combine high throughput screening assays with high content screening assays, all in the same well. First, a fast screen of all wells for the secreted bioluminescent reporter could identify potential hits. Then only the wells for those potential hits would need to be analyzed for more detailed information based on the high content screen. This would allow more efficient and detailed data mining of these wells, without compromising throughput.

We anticipate that the interest in live cell assays and their multiplexing capabilities will increase, due to the growing scientific interest in primary cells and stem cells. These cells are often only available in limited numbers [28, 29], due to their elaborate tissue culture requirements. Therefore it is imperative to develop multiplexing assays, in order to gain the maximum amount of data possible without sacrificing these precious cells.

The use of secreted reporter assays in stem cell research would, for example, allow for continuous monitoring of media supernatant from the same culture during subsequent differentiation stages. In the case of primary neuronal cells, live cell assays based on fluorescent proteins and/or secreted bioluminescent reporters could be developed to monitor the effect of repeated stimulation on receptor downregulation and desensitization in the same set of primary cells *via *continuous sampling. This type of long term monitoring experiment on valuable cells is only possible with live cell assays.

Although the reporters used in this work demonstrated very good performance in this set of applications, further improvements could enable even broader applications. For example, the signal decay rate of secreted *Metridia* luciferase to half of its original intensity is 10 minutes, which requires improvement. Although other bioluminescent reporters like *Gaussia* luciferase have considerably faster signal decay rates (in the range of 30–60 seconds) ^1^, the signal stability of *Metridia* luciferase activity will need to be further stabilized. This will be especially important when slower plate readers are used to analyze 384- or even 1536-well plates. If the signals from all wells are acquired simultaneously, fast signal decay is not problematic. However if acquisition occurs on a well-to-well basis, the signal intensity measured in the first and the last well would differ considerably.

Signal stabilization could be accomplished by a variety of approaches: Once the enzymatic site in secreted *Metridia* luciferase is identified, it may be possible to mutate this domain in order to change the enzymatic turnover rate of the substrate. Another possible approach could involve targeting the substrate itself. Changes in the structure of the substrate could lead to changes in the turnover rate, including association of the substrate with the luciferase and the dissociation of the product after the enzymatic reaction. Structural modifications to the substrate could also affect the intensity of the bioluminescent signal it produces upon interaction with the luciferase.

Further improvements also need to be made to fluorescent proteins for use in live cell reporter applications, in order to guarantee the lowest possible level of cytotoxicity. Minimizing cytotoxicity is especially important for applications in primary cells, stem cells, and animal models. Additionally, brighter, further red shifted fluorescent proteins must be developed to enhance live cell applications, due to their better signal to noise ratio when expressed *in vivo*.

Fluorescent proteins that act as sensors for specific events in a cell, either by changing their emission spectra or their fluorescence intensity, also need to be improved in order to create options for even more informative multiplexed assays, combining fluorescent, bioluminescent, and other assays such as absorbance based assays, all in the same live cell.

## Footnotes

1 New England Biolabs, Technical Bulletin E3300, *Gaussia* Luciferase Products 2007 October. Available from: http://www.neb.com/nebecomm/products/productE3300.asp.
